# Towards direct visualization of the reaction coordinates of proteins

**DOI:** 10.1063/4.0001050

**Published:** 2025-10-27

**Authors:** Doeke R Hekstra, BoRam Lee, Rama Ranganathan, Kristopher I White, Margaret A. Klureza, Jack B. Greisman, Kevin M. Dalton, Robert W Henning, Vukica Srajer

**Affiliations:** 1 Harvard University, Cambridge, MA, USA; 2 University of Chicago, Chicago, IL, USA; 3 Stanford University, Stanford, CA, USA; 4 D.E. Shaw Research, New York, NY, USA; 5 SLAC National Lab, Menlo Park, CA, USA; 6 New York University, New York, NY, USA

## Abstract

As for man-made machines, the functional cycles of enzymes and other proteins involve the coordinated motion of their parts. These motions, and the mechanical properties of proteins more generally, therefore link protein structure, function, and evolution. For many proteins, experimental access to protein mechanics remains elusive. I will describe our efforts to access these dynamics by electric field-stimulated X-ray crystallography and complementary methods. I will illustrate this approach using two examples: our work on the direct visualization of K+ ion permeation through an ion channel, and on the dissection of structural intermediates enzymatic cycle of dihydrofolate reductase.

